# Severe and/or prolonged COVID-19 in hematologic diseases: clinical implications before and during the omicron era

**DOI:** 10.3389/fonc.2025.1687204

**Published:** 2025-11-05

**Authors:** Akinao Okamoto, Masahiro Yoshida, Senji Kasahara, Takahide Ara, Kazutaka Ozeki, Takanobu Morishita, Daisuke Ikeda, Minoru Kanaya, Tomohiro Kajiguchi, Yasuhiro Suzuki, Shingo Kurahashi, Tomohiro Horio, Yoshiaki Marumo, Tatsuo Oyake, Shigeki Saito, Hitomi Sawa, Shun-ichi Kimura, Takahiro Nishiyama, Eisei Kondo, Junji Hiraga, Hiroki Hosoi, Yasufumi Masaki, Yoshiko Atsuta, Hideyuki Yamamoto, Takahiko Miyama, Naoe Goto, Chisako Iriyama, Keichiro Mihara, Yoshihiro Inamoto, Akihiro Tomita

**Affiliations:** ^1^ Department of Hematology, Fujita Health University School of Medicine, Toyoake, Japan; ^2^ Department of Hematology, Osaka City General Hospital, Osaka, Japan; ^3^ Department of Hematology, Gifu Municipal Hospital, Gifu, Japan; ^4^ Department of Hematology, Hokkaido University Hospital, Sapporo, Japan; ^5^ Department of Hematology and Oncology, JA Aichi Konan Kosei Hospital, Konan, Japan; ^6^ Department of Hematology, Japanese Red Cross Aichi Medical Center Nagoya Daiichi Hospital, Nagoya, Japan; ^7^ Department of Blood and Marrow Transplantation and Cellular Therapy, Fujita Health University School of Medicine, Toyoake, Japan; ^8^ Division of Hematology/Oncology, Department of Internal Medicine, Kameda Medical Center, Kamogawa, Japan; ^9^ Blood Disorders Center, Aiiku Hospital, Sapporo, Japan; ^10^ Department of Hematology and Oncology, Tosei General Hospital, Seto, Japan; ^11^ Department of Hematology, National Hospital Organization Nagoya Medical Center, Nagoya, Japan; ^12^ Department of Hematology and Oncology, Toyohashi Municipal Hospital, Toyohashi, Japan; ^13^ Division of Hematology, Department of Internal Medicine, Aichi Medical University School of Medicine, Nagakute, Japan; ^14^ Department of Hematology and Oncology, Nagoya City University, Nagoya, Japan; ^15^ Division of Hematology and Oncology, Department of Internal Medicine, Iwate Medical University School of Medicine, Morioka, Japan; ^16^ Department of Hematology and Oncology, Japanese Red Cross Aichi Medical Center Nagoya Daini Hospital, Nagoya, Japan; ^17^ Department of Hematology and Oncology, Anjo Kosei Hospital, Anjo, Japan; ^18^ Division of Hematology, Jichi Medical University Saitama Medical Center, Saitama, Japan; ^19^ Division of Hematology, Ichinomiya Municipal Hospital, Ichinomiya, Japan; ^20^ Department of Hematology, Kawasaki Medical School, Kurashiki, Japan; ^21^ Department of Hematology, Toyota Kosei Hospital, Toyota, Japan; ^22^ Department of Hematology/Oncology, Wakayama Medical University, Wakayama, Japan; ^23^ Department of Hematology and Immunology, Kanazawa Medical University, Kanazawa, Japan; ^24^ Japanese Data Center for Hematopoietic Cell Transplantation, Nagakute, Japan; ^25^ International Center for Cell and Gene Therapy, Fujita Health University, Toyoake, Japan

**Keywords:** COVID-19, hematologic diseases, Omicron variant, prognosis, immunocompromised patients, multicenter study

## Abstract

**Background:**

Although the Omicron variant has been reported to reduce COVID-19 severity in the general population, its impact on patients with hematologic malignancies remains uncertain, and epidemiological investigation is warranted.

**Methods:**

We conducted a multicenter retrospective cohort study of 1, 023 patients with hematologic diseases diagnosed with COVID-19 at 22 centers in Japan between January 2020 and January 2023. Outcomes within 60 days after diagnosis including severe and/or prolonged disease, COVID-19–related mortality, and overall survival (OS) were compared between the pre-Omicron and Omicron periods. Multivariable analysis was performed to identify independent adverse prognostic factors.

**Results:**

Severe and/or prolonged disease occurred in 27.5% of patients, COVID-19–related mortality was 6.3%, and OS was 91.4%. Compared with the pre-Omicron period, the Omicron period was associated with significantly lower rates of severe/prolonged disease (26.0% vs. 48.0%, *P<*0.01) and COVID-19–related mortality (5.0% vs. 15.0%, *P<*0.01), but no significant difference in OS (92.0% vs. 84.0%, *P* = 0.62). Age ≥60 years was the strongest predictor of severe/prolonged disease (sHR 3.08, *P<*0.01) and mortality (HR 8.94, *P<*0.01). Male sex (sHR 1.38; HR 1.82, both *P<*0.01) and prior bendamustine exposure (sHR 1.83; HR 1.87, both *P<*0.01) were also associated with both outcomes, whereas anti-CD38 antibody therapy was linked only to mortality (HR 3.65, *P<*0.01).

**Conclusion:**

In patients with hematologic diseases, the Omicron period was associated with reduced severity and COVID-19–related mortality but no improvement in OS. Older age and prior bendamustine exposure were strongly associated with adverse outcomes, highlighting the need for strict infection prevention and prompt, aggressive COVID-19 management in these high-risk populations.

## Introduction

The epidemiology of coronavirus disease 2019 (COVID-19) has evolved over time. In Japan, the Omicron variant became predominant in January 2022, leading to three subsequent waves during the summer and winter of that year (1, 2). The Omicron variant has shown increased transmissibility in the general population (3, 4), although its associated mortality rate has declined (5–7). However, several reports have suggested that the Omicron variant might have a more profound impact on patients with hematologic diseases than in the general population (8–13). Recent large real-world registry studies, including the EPICOVIDEHA analysis in multiple myeloma and the Spanish GETH-TC registry in hematologic malignancies, have further confirmed that immunocompromised patients remain at considerable risk for severe and fatal COVID-19 even during the Omicron era (14, 15). Significant numbers of patients in the Omicron era still have severe disease requiring oxygen supplementation or admission to an intensive care unit, and COVID-19 has been fatal in some patients previously treated with bendamustine-containing regimens (13, 16). Others have had prolonged disease, with persistently positive SARS-CoV-2 viral loads requiring continued isolation in the hospital (13, 16, 17).

Many more therapeutic agents against SARS-CoV-2 became clinically available during the Omicron era than in earlier periods (18). Although the prognosis for patients is expected to improve, severe and/or prolonged and fatal cases are still being experienced in clinical practice. Several reports have indicated that prolonged disease may facilitate additional genetic mutations, leading to resistance against antiviral agents such as the anti-SARS-CoV-2 antibodies casirivimab/imdevimab, sotrovimab, and tixagevimab/cilgavimab (19–21); and to small molecules such as remdesivir (13, 22) and nirmatrelvir (23), potentially exacerbating disease severity and duration (13, 22, 24, 25). These phenomena suggest that in the clinical setting, it is critically important to identify the patient groups most vulnerable to severe and/or prolonged disease.

In addition, with the advent of the Omicron variant, multiple vaccines have become available and the situation has markedly changed compared to the early phase of the pandemic. However, patients with hematologic malignancies undergoing specific treatments, such as anti-CD20 antibodies and Bruton’s tyrosine kinase inhibitors, have exhibited markedly impaired humoral responses to COVID-19 vaccination (26–30). The extent to which immunodeficiency contributes to the risk of developing severe and/or prolonged disease, or mortality, during the Omicron era remains uncertain. Furthermore, as with antiviral drugs, especially in the Omicron era, reductions in the efficacy of both monovalent and bivalent vaccines due to additional mutations in the viral genome have also been reported (31–34). These factors are also considered to have a significant impact on patients with hematologic diseases.

Given the special circumstances of immunosuppression and the use of antitumor drugs in patients with hematologic diseases, we conducted a survey of actual clinical practice to assess whether the effects of COVID-19 in the Omicron era may be different in these patients than in the general population. The aim of this multicenter, large-scale retrospective cohort study was to evaluate the clinical outcomes of COVID-19 in patients with hematologic diseases, with a particular focus on the clinically significant composite event of severe disease requiring oxygen therapy and/or prolonged disease requiring isolation in the hospital for more than 3 weeks. These findings may help identify patients at high risk for severe COVID-19 who may require more intensive infection control measures and antiviral interventions during the Omicron era.

## Methods

### Patients

**Figure 1:**
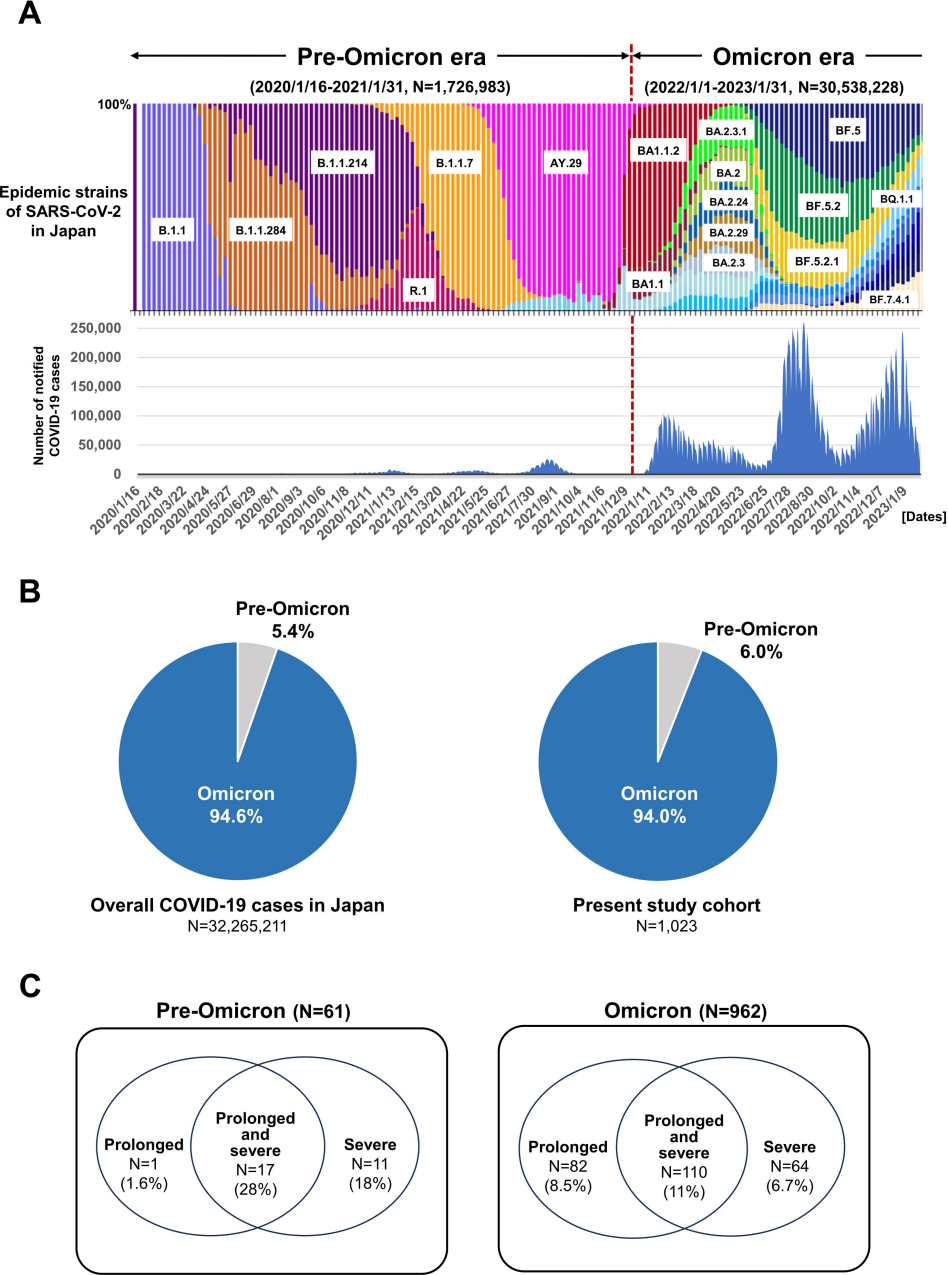
Trends in cases of COVID-19 during the pre-Omicron and Omicron eras in Japan. **(A)** Colored stacked bar graph shows nationwide trends in SARS-CoV-2 epidemic strains (upper panel). The corresponding numbers of COVID-19 patients are indicated by blue bars (lower panel). **(B)** Proportion of overall COVID-19 patients during the pre-Omicron and Omicron eras in Japan (left panel) and in the present study cohort (right panel). **(C)** Proportions of the present COVID-19 patients with prolonged, prolonged and severe, and severe disease during the pre-Omicron (left panel) and Omicron eras (right panel).

This multicenter retrospective study included patients who received treatment for hematologic diseases at 22 hospitals in Japan between January 1, 2020 and January 31, 2023, and who were newly diagnosed with SARS-CoV-2 infection by genetic testing (polymerase chain reaction) or antigen testing using nasal swab or saliva. Based on data from the Ministry of Health, Labour and Welfare and the National Institute of Infectious Diseases in Japan, the pre-Omicron era was defined as that up to December 31, 2021, and the Omicron era as beginning on January 1, 2022 (**Figure 1A**). Patients who had been diagnosed at institutions other than the participating centers and whose diagnoses were not confirmed at the study institutions were excluded. Clinical data were collected from electronic medical records using a structured research questionnaire. Due to the observational nature of the study, patient consent to participate was not required. However, information about the study was disclosed on each institution’s website, and patients could choose to opt out. The study was approved by the Ethics Review Committee of Fujita Health University School of Medicine (approval numbers: HM-23-010, HM-23-082) and affiliated institutions and was conducted according to the principles of the Declaration of Helsinki.

### Definitions

The primary endpoint was the cumulative incidence of patients who experienced severe and/or prolonged disease within 60 days of COVID-19 diagnosis. The secondary endpoints were the overall survival rate at day 60 and COVID-19 related mortality. COVID-19 related mortality was defined as mortality during COVID-19 positivity or due to COVID-19-related complications. Risk factors associated with each endpoint were also analyzed. Severe disease was defined as COVID-19 requiring oxygen supplementation or admission to an intensive care unit. Prolonged disease was defined as continued isolation in hospital beyond 21 days after symptom onset, based on the attending physician’s judgment, due to persistent positivity on genetic or antigen testing or ongoing clinical symptoms. The hematologic diseases included in this study were classified into lymphoid malignancies, myeloid malignancies, and other benign hematologic disorders. To account for the potential impact of treatment regimens, lymphoid malignancies were further subdivided into B-cell lymphomas, plasma cell neoplasms, and other lymphoid malignancies for separate analyses (**Table 1**).

**Table 1:** Characteristics of the study patients.

Characteristic, n (%)	Total	Pre-omicron era	Omicron era	*P*-value
Total	1.023	61	962	
Sex				
Male	613(60)	36 (59)	577 (60)	*0.89*
Female	410 (40)	25 (41)	385 (40)	
Median age, years (range)	69 (13-97)	68 (20-92)	69 (13-97)	*0.90*
Diagnosis				
Myeloid malignancies	257 (25)	17 (28)	240 (25)	*0.54*
AML	74	7	67	
MDS	88	3	85	
CML	55	6	49	
MPN other than CML	38	1	37	
Other myeloid malignancies	2	0	2	
Lymphoid malignancies	617 (60)	32 (52)	585 (61)	*0.22*
ALL	58	4	54	
DLBCL	186	11	175	
FL	106	3	103	
HL	16	2	14	
Indolent B-lymphoma, others*	77	4	73	
T/NK cell lymphoma	61	3	58	
Plasma cell neoplasms	98	5	93	
Other lymphoid malignancies	15	0	15	
Benign hematologic disorders	153(15)	13 (21)	140 (15)	*0.36*
ITP	55	6	49	
AA	10	2	8	
AIHA	7	1	6	
Others	81	4	77	
Treatment				
Bendamustine	113 (11)	1 (2)	112 (12)	*0.010*
Anti-CD20 Abs	284 (28)	8 (13)	276 (29)	*0.039*
BTK inhibitors	28 (3)	3 (5)	25 (3)	*0.23*
Polatuzumab-vedotin	18 (2)	0 (0)	18 (2)	*0.62*
Anti-CD38 Abs	52 (5)	4 (6)	48 (5)	*0.54*
JAK2 inhibitors	10 (1)	1 (2)	9 (1)	*0.46*
Allogeneic SCT	44 (4)	2 (3)	42 (4)	*1.0*
Autologous SCT	26 (3)	2 (3)	24 (2)	*1.0*
CAR-T	9 (1)	0 (3)	9 (1)	*1.0*

AML, acute myelogenous leukemia; MDS, myelodysplastic syndromes; MPN, myeloproliferative neoplasms; CML, chronic myelocytic leukemia; DLBCL, diffuse large B-cell lymphoma; Follicular lymphoma; HL, Hodgkin lymphoma; ITP; immune thrombocytopenia; AA, aplastic anemia; AIHA, autoimmune hemolytic anemia; BTK, Bruton’s tyrosine kinase; JAK2, Janus kinase 2; SCT, stem cell transplantation; CAR-T, chimeric antigen receptor T-cell therapy. *Indolent lymphomas including marginal zone lymphoma; mucosa-associated lymphoid tissue lymphoma; chronic lymphocytic leukemia/small lymphocytic lymphoma; hairy cell leukemia; mantle cell lymphoma and lymphoplasmacytic lymphoma.

### Statistical analysis

Fisher’s exact test was used to compare categorical variables between groups. The cumulative incidence of severe and/or prolonged disease was calculated from the date of COVID-19 diagnosis, treating death without severe or prolonged disease as a competing risk event. Gray’s test was used to compare the cumulative incidence of COVID-19-related mortality between groups. Cumulative incidence was calculated from the date of COVID-19 diagnosis, treating deaths due to causes other than COVID-19 as competing risks. Overall survival was calculated using the Kaplan–Meier method and was compared using the log-rank test.

Fine–Gray proportional hazards regression models were used for analyses of risk factors associated with severe and/or prolonged disease and COVID-19 related mortality. Cox proportional hazards regression models were used for analyses of factors associated with overall mortality. Factors with a *p* value<0.05 in univariate analysis were entered into multivariate analysis, and a backward elimination procedure was used in developing final models, using a *p* value threshold of 0.05. A two-sided *p* value of less than 0.05 was considered statistically significant. During the preparatory meeting prior to the initiation of the study, it was concluded based on clinical experience that bendamustine, anti-CD20 antibody therapy, allogeneic hematopoietic cell transplantation (allo-HCT), and autologous hematopoietic cell transplantation may result in prolonged immunosuppression lasting more than one year. Accordingly, treatment history within the past two years was considered for these therapies. In contrast, therapies such as anti-CD38 antibody therapy, Bruton’s tyrosine kinase inhibitors, Janus kinase inhibitors, and chimeric antigen receptor (CAR) T-cell therapy were evaluated based on treatment history within the past one year.

All statistical analyses were performed using EZR (Jichi Medical University Saitama Medical Center, Saitama, Japan; http://www.jichi.ac.jp/saitama-sct/SaitamaHP.files/statmedEN.html), a graphical user interface for R (The R Foundation for Statistical Computing, Vienna, Austria) (35).

## Results

### Patients

Based on the data from the Japanese Ministry of Health, Labour and Welfare and the National Institute of Infectious Diseases, there was a significant accumulation of COVID-19 cases associated with the Omicron variant after January 2023, with a wide variety of SARS-CoV-2 sub-lineages detected (**Figure 1A**) (1, 2). A total of 1, 023 COVID-19 patients with hematologic diseases were enrolled in this multicenter study (**Table 1**). The median age was 69 years (range, 13–97 years). Among them, 61 patients developed COVID-19 during the pre-Omicron era, and 962 during the Omicron era. The distribution of case numbers across the two eras was consistent with previously reported national data (**Figure 1B**). There were no significant differences in sex or age distribution, or in the distribution of underlying hematologic diseases, between the pre-Omicron and Omicron eras. A significant difference was observed in terms of treatment history. The frequency of use of drugs such as bendamustine (*P* = 0.010) and anti-CD20 antibodies (*P* = 0.039) was significantly higher during the Omicron era. Other treatment strategies, including Bruton’s tyrosine kinase inhibitors, anti-CD38 antibodies, Janus kinase 2 inhibitors, allogeneic and autologous SCT, and CAR-T therapy, were used at similar frequencies in both eras. These findings indicate that although baseline characteristics and disease distributions were generally similar, the use of bendamustine and anti-CD20 antibodies may have changed in the clinical setting, likely reflecting changes in the SARS-CoV-2 epidemic status and treatment modalities.

### Incidence of severe and/or prolonged disease

Among the 1, 023 COVID-19 patients included in this study, a total of 281 (27.5%) met the criteria for severe and/or prolonged COVID-19 within 60 days (95% confidence interval (CI): 25.0%–30.1%). Of these, 29 cases (48.0%) occurred during the pre-Omicron period, and 252 cases (26.0%) during the Omicron period. The distribution of these cases by period is shown in **Figure 1C**.

**Figure 2:**
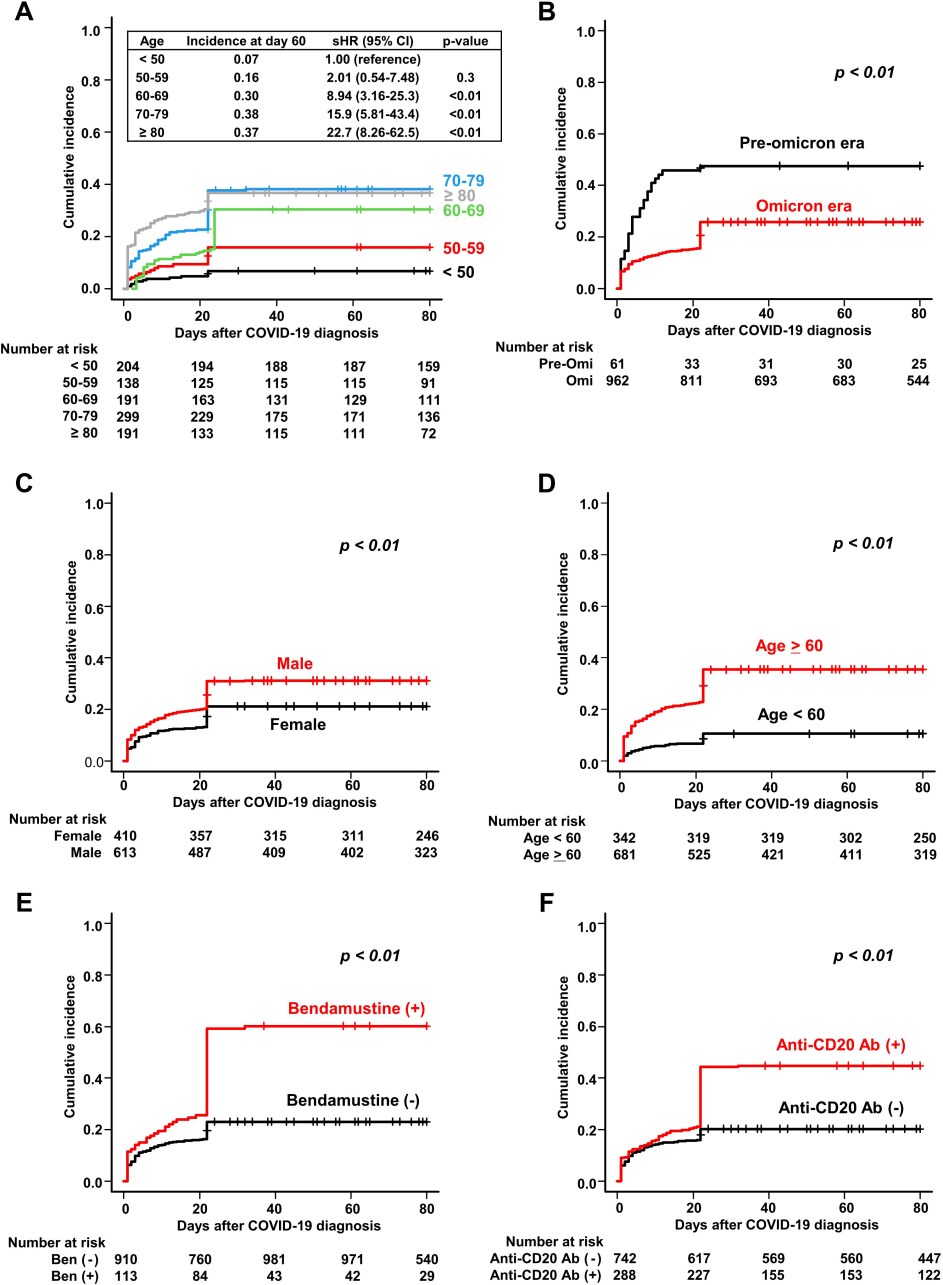
Cumulative incidence of severe and/or prolonged COVID-19 in patients with hematologic diseases. Cumulative incidence of severe and/or prolonged disease is stratified by **(A)** age, **(B)** time period of COVID-19 onset (pre-Omicron and Omicron), **(C)** sex, **(D)** age (≥60 and <60 years), **(E)** bendamustine use, and **(F)** anti-CD20 antibody use.

The cumulative incidence of severe and/or prolonged disease within 60 days after COVID-19 diagnosis (the primary endpoint) was 27.5% (95% CI, 25.0%–30.1%) (**Table 2**). As expected, the incidence increased as patient age increased, with 60 years considered an appropriate cut-off age for subsequent analysis (**Figure 2A**). **Table 2** shows the results of regression analyses conducted to identify factors associated with severe and/or prolonged disease within 60 days. Univariate analysis identified pre-Omicron era, male sex, age ≥ 60 years, B-cell lymphoma, plasma cell neoplasms, bendamustine use, anti-CD20 antibody use, and polatuzumab vedotin use as statistically significant factors associated with severe and/or prolonged disease (*P<*0.01 for all). Multivariate analysis identified pre-Omicron era (sHR 2.32, 95% CI 1.56–3.45, *P<*0.01), male sex (sHR 1.38, 95% CI 1.08–1.75, *P<*0.01), age ≥ 60 years (sHR 3.08, 95% CI 2.16–4.39, *P<*0.01), bendamustine therapy (sHR 1.83, 95% CI 1.37–2.44, *P<*0.01), and anti-CD20 antibody therapy (sHR 1.48, 95% CI 1.12–1.95, *P<*0.01) as statistically significant factors associated with severe and/or prolonged disease. **Figures 2B–F** show cumulative incidence curves according to the presence or absence of these individual factors. These findings suggest that specific types of hematologic diseases and therapeutic strategies, in addition to older age and male sex, significantly correlate with the incidence of severe and/or prolonged disease.

**Table 2:** Univariate and multivariate analysis of factors associated with severe and/or prolonged diseases.

Covariate	N	Incidence at day 60 (95% CI)	Univariate		Multivariate	
			sHR (95% CI)	*P*-value	sHR (95% CI)	*P*-value
All	1023	0.27 (0.25-0.30)				
Era						
Omicron	962	0.26 (0.23-0.29)	1.00 (reference)		1.00 (reference)	
Pre-omicron	61	0.48 (0.35-0.60)	2.32 (1.56-3.45)	*<0.01*	2.78 (1.82-4.17)	*<0.01*
Sex						
Female	410	0.21 (0.17-0.25)	1.00 (reference)		1.00 (reference)	
Male	613	0.31 (0.28-0.35)	1.53 (1.20–1.95)	*<0.01*	1.38 (1.08–1.75)	*<0.01*
Age						
<60 years	342	0.11 (0.08-0.14)	1.00 (reference)		1.00 (reference)	
≥60 years	681	0.36 (0.32-0.39)	3.82 (2.70–5.39)	*<0.01*	3.08 (2.16–4.39)	*<0.01*
Diagnosis						
Benign hematologic disorders	156	0.13 (0.08-0.19)	1.00 (reference)			
Myeloid malignancies	246	0.18 (0.13-0.23)	1.42 (0.83-2.42)	*0.20*		
B-cell lymphomas	400	0.39 (0.35-0.44)	3.31 (2.08–5.29)	*<0.01*		
Plasma cell neoplasms	97	0.31 (0.22-0.40)	2.62 (1.49-4.63)	*<0.01*		
Other lymphoid malignancies	124	0.22 (0.15-0.29)	1.71 (0.97-3.05)	*0.07*		
Treatment						
Bendamustine	113	0.60 (0.51-0.69)	1.87 (1.40-2.48)	*<0.01*	1.83 (1.37–2.44)	*<0.01*
Anti-CD20 Abs	288	0.49 (0.39-0.50)	1.74 (1.33–2.28)	*<0.01*	1.48 (1.12- 1.95)	*<0.01*
Anti-CD38 Abs	52	0.35 (0.22-0.48)	1.42 (0.89–2.27)	*0.14*		
BTK inhibitors	28	0.43 (0.24-0.60)	1.78 (0.98–3.25)	*0.06*		
JAK2 inhibitors	10	0.20 (0.03-0.50)	0.75 (0.20–2.78)	*0.67*		
Polatuzumab-vedotin	18	0.56 (0.29-0.75)	2.75 (1.46–5.18)	*<0.01*		
Allogeneic HCT	44	0.18 (0.08-0.31)	0.62 (0.30–1.23)	*0.17*		
Autologous HCT	26	0.27 (0.12-0.45)	0.79 (0.37–1.70)	*0.55*		
CAR-T	9	0.56 (0.12-0.82)	2.23 (0.94–5.59)	*0.07*		

sHR, subdistribution hazard ratio; BTK, Bruton’s tyrosine kinase; CI, confidence interval; JAK2, Janus kinase 2; HCT; hematopoietic cell transplantation; CAR-T, chimeric antigen receptor T-cell therapy.

### Overall survival

**Figure 3:**
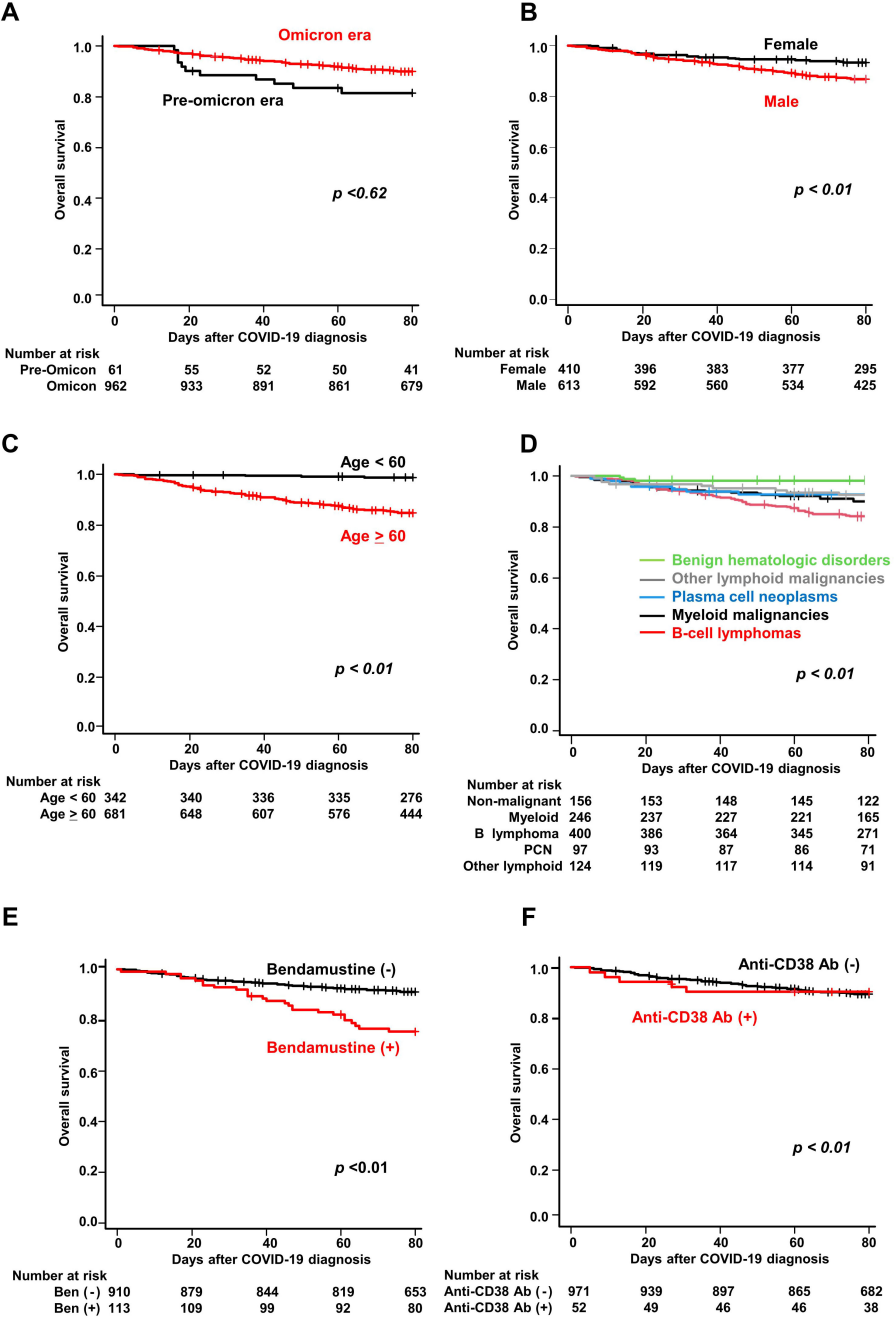
Overall survival of COVID-19 patients with hematologic diseases. Kaplan–Meier curves of overall survival stratified by **(A)** time period of COVID-19 onset (pre-Omicron and Omicron), **(B)** sex, **(C)** age (≥60 vs <60 years), **(D)** hematologic disease group, **(E)** bendamustine use, and **(F)** anti-CD38 antibody use.

The probability of overall survival at 60 days after COVID-19 diagnosis was 91.4% (95% CI, 89.6%–92.9%) (**Table 3**). The probabilities of overall survival were similar during the pre-Omicron and Omicron periods (84.0% and 92.0%, respectively, *P*<0.62, **Figure 3A**, **Table 3**). Univariate analysis identified male sex, age ≥60 years, myeloid malignancies, B-cell lymphoma, other lymphoid malignancies, bendamustine use, anti-CD20 antibody use, and anti-CD38 antibody use as statistically significant risk factors (*P*<0.01 for all). Multivariate analysis identified male sex (HR, 1.82; 95% CI, 1.29–2.58; *P*<0.01), age ≥60 years (HR, 8.94; 95% CI, 4.52–17.70; *P*<0.01), myeloid malignancies (HR, 3.77; 95% CI, 1.49–9.55; *P*<0.01), B-cell lymphoma (HR, 3.16; 95% CI, 1.26–7.92; *P* = 0.01), other lymphoid malignancies (HR, 3.95; 95% CI, 1.45–10.7; *P<*0.01), prior bendamustine use (HR, 1.87; 95% CI, 1.23–2.84; *P*<0.01) and prior anti-CD38 antibody use (HR, 3.65; 95% CI, 1.55–8.59; *P*<0.01) as independently associated with overall survival. **Figures 3B–F** show survival curves according to the presence or absence of these individual factors.

**Table 3:** Univariate and multivariate analysis for overall survival.

Covariate	N	Survival at day 60 (95% CI)	Univariate		Multivariate	
			HR (95% CI)	*P*-value	HR (95% CI)	*P*-value
All	1023	0.91 (0.90-0.93)				
Era						
Omicron	962	0.92 (0.90-0.94)	1.00 (reference)	*0.62*		–
Pre-omicron	61	0.84 (0.72-0.91)	1.15 (0.67-2.00)			
Sex						
Female	410	0.95 (0.92-0.96)	1.00 (reference)		1.00 (reference)	
Male	613	0.89 (0.87-0.92)	2.2 (1.56–3.10)	*<0.01*	1.82 (1.29–2.58)	*<0.01*
Age						
<60 years	342	0.99 (0.97-1.00)	1.00 (reference)		1.00 (reference)	
≥60 years	681	0.88 (0.85-0.90)	11.1 (5.67-21.7)	*<0.01*	8.94 (4.52–17.7)	*<0.01*
Diagnosis						
Benign hematologic disorders	156	0.98 (0.94-099)	1.00 (reference)		1.00 (reference)	
Myeloid malignancies	246	0.92 (0.88-0.95)	6.13 (2.43-15.5)	*<0.01*	3.77 (1.49-9.55)	*<0.01*
B cell lymphomas	400	0.87 (0.84-0.90)	7.90 (3.21-19.4)	*<0.01*	3.16 (1.26-7.92)	*0.01*
Plasma cell neoplasms	97	0.93 (0.85-0.97)	6.96 (2.61-18.6)	*<0.01*	1.56 (0.48-5.10)	*0.45*
Other lymphoid malignancies	124	0.94 (0.87-0.97)	4.49 (1.66-12.2)	*<0.01*	3.95 (1.45-10.7)	*<0.01*
Treatment						
Bendamustine	113	0.82 (0.74-0.88)	2.51 (1.75–3.61)	*<0.01*	1.87 (1.23–2.84)	*<0.01*
Anti-CD20 Abs	288	0.88 (0.83-0.91)	1.82 (1.35–2.45)	*<0.01*		
Anti-CD38 Abs	52	0.90 (0.78-0.96)	2.15 (1.30–3.55)	*<0.01*	3.65 (1.55- 8.59)	*<0.01*
BTK inhibitors	28	0.85 (0.65-0.94)	1.35 (0.63–2.88)	*0.43*		
JAK2 inhibitors	10	0.90 (0.47-0.99)	1.27 (0.32–5.14)	*0.67*		
Polatuzumab-vedotin	18	0.78 (0.51-0.91)	2.30 (1.02–5.20)	*0.04*		
Allogeneic HCT	44	0.98 (0.85-1.00)	0.57 (0.23–1.38)	*0.21*		
Autologous HCT	26	0.96 (0.76-0.99)	0.64 (0.21–2.01)	*0.45*		
CAR-T	9	1.00 (NA)	NA	NA		

HR, Hazard ratio; BTK, Bruton’s tyrosine kinase; CI, confidence interval; JAK2, Janus kinase 2; HCT, hematopoietic cell transplantation; CAR-T, chimeric antigen receptor T-cell therapy; NA; not applicable.

### Incidence of COVID-19 related mortality

The cumulative incidence of COVID-19-related mortality was 6.3% (95% CI, 5.0%–7.7%) at 60 days after COVID-19 diagnosis (**Table 4**). The incidence was significantly lower in the Omicron era than that in the pre-Omicron era (5.0% vs. 15.0%, *P*<0.01). **Table 4** lists the factors associated with COVID-19 related mortality. Univariate analysis identified pre-Omicron era, male sex, age ≥60 years, B-cell lymphoma, bendamustine use, anti-CD20 antibody use, and polatuzumab vedotin use as factors associated with increased COVID-19 mortality. In multivariate analysis, age ≥60 years was the strongest independent predictor of COVID-19 related mortality (9.0% vs. 1.0%; sHR, 11.47; 95% CI, 3.62–36.33; *P*<0.01). Prior bendamustine use was also independently associated with significantly higher mortality (14.0%; sHR, 3.67; 95% CI, 2.33–5.77; *P*<0.01) (**Table 4**). Male sex and B-cell lymphoma were not retained in the multivariate model. No deaths were observed among patients who underwent allogeneic hematopoietic cell transplantation (n = 44) or CAR-T cell therapy (n = 9) during the observation period. Among patients in the present cohort who died, the proportion of deaths classified as COVID-19–related was approximately 90.0% (9/10) during the pre-Omicron era, which decreased to approximately 65.4% (51/78) during the Omicron era. These data suggest that although specific types of hematologic diseases and therapeutic strategies are also significantly correlated with COVID-19-related mortality, the risk of mortality due to COVID-19 showed a decreasing tendency in the Omicron era compared with that in the pre-Omicron era.

**Table 4:** Univariate and multivariate analysis for COVID-19-related mortality.

Covariate	N	Incidence at day 60 (95% CI)	Univariate		Multivariate	
			sHR (95% CI)	*P*-value	sHR (95% CI)	*P*-value
All	1023	0.06 (0.05-0.08)				
Era						
Omicron	962	0.05 (0.04-0.07)	1.00 (reference))		1.00 (reference)	
Pre-omicron	61	0.15 (0.07-0.25)	2.08 (1.08-4.17)	*<0.01*	2.63 (1.30-5.26)	*<0.01*
Sex						
Female	410	0.04 (0.03-0.06)	1.00 (reference)		–	
Male	613	0.07 (0.05-0.09)	1.90 (1.17–3.10)	*0.01*	–	
Age						
<60 years	342	0.01 (0.00-0.02)	1.00 (reference)		1.00 (reference)	
≥60 years	681	0.09 (0.07-0.11)	14.2 (4.48-45.0)	*<0.01*	11.5 (3.62–36.3)	*<0.01*
Diagnosis						
Benign hematologic disorders	156	0.02 (0.01-0.05)	1.00 (reference)		–	
Myeloid malignancies	246	0.05 (0.02-0.08)	3.06 (0.87-10.7)	*0.08*	–	
B cell lymphomas	400	0.09 (0.07-0.12)	7.43 (2.31-23.9)	*<0.01*	–	
Plasma cell neoplasms	97	0.04 (0.01-0.10)	2.13 (0.47-9.60)	*0.33*	–	
Other lymphoid malignancies	124	0.05 (0.02-0.10)	2.50 (0.62-10.2)	*0.19*	–	
Treatment						
Bendamustine	113	0.14 (0.04-0.06)	4.53 (2.86–7.15)	*<0.01*	3.67 (2.33–5.77)	*<0.01*
Anti-CD20 Abs	288	0.09 (0.06-0.12)	2.65 (1.72–4.08)	*<0.01*	–	–
Anti-CD38 Abs	52	0.06 (0.02-0.12)	0.74 (0.23–2.35)	*0.61*	–	–
BTK inhibitors	28	0.15 (0.05-0.31)	2.23 (0.90–5.52)	*0.08*	–	–
JAK2 inhibitors	10	0.10 (0.01-0.37)	1.31 (0.18–9.40)	*0.79*	–	–
Polatuzumab-vedotin	18	0.17 (0.04-0.07)	2.30 (1.02–5.20)	*0.04*	–	–
Allogeneic HCT	44	NA	NA		–	–
Autologous HCT	26	0.04 (0.00-0.17)	0.46 (0.06–3.31)	*0.44*	–	–
CAR-T	9	NA	NA		–	–

sHR, subdistribution hazard ratio; BTK, Bruton’s tyrosine kinase; CI, confidence interval; JAK2, Janus kinase 2; HCT; hematopoietic cell transplantation; CAR-T, chimeric antigen receptor T-cell therapy; NA, not applicable.

### Post-COVID-19 complications

Post-COVID-19 complications were confirmed in 79 patients (8.5%), of whom 57 had respiratory complications, the most frequent of which was interstitial pneumonia (n = 42) (**Supplementary Table S1**). Among patients with interstitial pneumonia as a post-COVID-19 complication, bendamustine was used in 13 (31%) (*P*<0.01, 95% CI 1.82–8.13, odds ratio 3.94). Severe fatigue, a common symptom of long COVID (36), occurred in five patients (0.5%).

## Discussion

This study demonstrates that even during the Omicron era, patients with hematologic malignancies remained at substantial risk for severe and/or prolonged COVID-19 and death. Multivariate analysis identified age >65 years and bendamustine use as major risk factors for adverse outcomes, whereas male sex and anti-CD38 antibody therapy were associated with increased overall mortality. These findings are consistent with earlier studies that have reported higher risks among older adults and male patients in the pre-Omicron era (37).

The proportion of deaths due to COVID-19 among all deaths decreased from 89.7% in the pre-Omicron era to 65.4% during the Omicron era. Decreasing virulence of Omicron strains and improvements in COVID-19 treatment strategies may be considered as possible reasons for this trend. However, the lack of a direct correlation between lower proportion of deaths due to COVID-19 and an improvement in overall survival suggests that indirect effects, such as clinical deterioration or delays in treatment for hematologic diseases, may have also contributed to mortality. These findings highlight the importance of considering both direct and indirect consequences of COVID-19 in managing immunocompromised patients.

It is important to note that prolonged disease beyond 60 days after COVID-19 diagnosis can occur in immunocompromised patients with hematologic diseases. In particular, patients with a history of anti-CD20 antibody and bendamustine combination therapy have been reported to exhibit persistent infections lasting 3–6 months or longer (13, 16, 38, 39). In such cases, persistent SARS-CoV-2 infection may facilitate the accumulation of genetic mutations in the viral genome, leading to resistance to antiviral drugs. Furthermore, secondary bacterial and/or fungal infections in the setting of severe immunodeficiency also represent serious complications in these patients (13, 22, 40). These findings strongly suggest the necessity of intensive management not only for prolonged SARS-CoV-2 infections but also for prevention and treatment of other infectious diseases.

This study demonstrated that five factors (older age, male sex, hematologic disease type, prior bendamustine use, and anti-CD38 antibody use) were significantly associated with poorer overall survival. Among these, age emerged as the most prominent predictor, indicating that patients aged 60 years or older with additional adverse prognostic factors may be critical candidates for timely antiviral therapy and intensive monitoring. Given the strong association between bendamustine use and adverse outcomes such as severe disease and mortality, its use warrants careful consideration, particularly in older or immunocompromised patients, when clinically appropriate. In COVID-19 patients with a history of bendamustine use, early intervention and ongoing close follow-up may support recovery from SARS-CoV-2 infection and reduce the risk of other infectious diseases. A risk-adapted, individualized management approach may prove beneficial not only during the Omicron era but also in the future, even if the virulence of SARS-CoV-2 decreases.

The impact of bendamustine on COVID-19 outcomes remains under-investigated, although several reports have suggested associations with severe and/or prolonged disease (41–43). Bendamustine can cause prolonged lymphocytopenia, particularly of CD4+ T cells (44–47), and has been linked to other viral infections, such as cytomegalovirus (48). Reduced vaccine responsiveness in patients receiving bendamustine has been also demonstrated (49). A recent multicenter retrospective study from Spain showed that patients with follicular lymphoma and mantle cell lymphoma who received bendamustine induction followed by rituximab maintenance had a higher risk of COVID-19 related death compared to those treated with cyclophosphamide-based regimens (50). Furthermore, Iriyama et al. and others have reported prolonged and/or severe COVID-19 in patients with marked reductions in CD19+ B-cells and CD4+ T-cells, and an increased proportion of exhausted CD4+ T-cells following treatment with bendamustine combined with anti-CD20 antibodies (13, 16). These findings may suggest that transient but prolonged impairment of T-cell function induced by bendamustine may in part contribute to deficiency of SARS-CoV-2 clearance. Recent reports, together with our findings, suggest that the use of bendamustine should be carefully evaluated, particularly in elderly or immunocompromised patients. In such patients, prompt initiation of antiviral therapy may be advisable immediately upon COVID-19 diagnosis.

In the present study, anti-CD20 antibody use was significantly associated with an increased risk of severe and/or prolonged COVID-19, but not with overall mortality. One possible explanation for this finding is the advancement of various therapeutic strategies for COVID-19. Another may be the limited impact of anti-CD20 antibody therapy on cellular immunity, which plays a critical role in viral clearance. We have previously demonstrated that treatment with anti-CD20 antibodies temporarily impairs the humoral immune response to SARS-CoV-2 vaccination, with anti-SARS-CoV-2 spike protein antibody titers remaining largely undetectable for approximately 9–12 months after the last administration. This phenomenon is closely associated with delayed B-cell recovery (26). During the acute phase of SARS-CoV-2 infection, cytotoxic responses mediated by CD8+ T cells are induced within 1 to 2 weeks of symptom onset and are known to contribute substantially to viral control and milder clinical outcomes (51–54). In addition to the importance of viral clearance, chronic inflammation driven by cellular immunity may play a critical role in the development of severe and/or prolonged disease and death. In this context, T-cell function may be particularly important. As anti-CD20 antibody treatment has minimal impact on cellular immune function, its use may have had little impact on patient survival.

Although allo-HCT is generally considered a high-risk factor for infection (55), there was no significant association of allo-HCT with severe infection or a higher mortality rate in our cohort. Immune reconstitution and improved vaccine responses in transplant recipients may contribute to reduced COVID-19 severity (56), suggesting that allo-HCT recipients may no longer be a high-risk population in the Omicron era, as previously reported (57, 58).

Limitations of this study include the absence of detailed information on COVID-19 treatment and its effect on clinical outcomes. In addition, selection bias may be present, as the analysis included only patients treated at participating hospitals that submitted data, potentially excluding milder cases that were managed at non-participating or community-based facilities. Information bias due to the retrospective data collection and unmeasured confounding factors, such as vaccination status, antiviral treatment, and degree of immunosuppression, also warrant consideration. Future research should incorporate standardized treatment data and evaluate the effectiveness of antiviral interventions in patients with hematologic diseases.

In conclusion, despite the milder nature of the Omicron variant, patients with certain types of hematologic diseases and specific conditions remain at risk for severe and/or prolonged COVID-19. Particular attention should be given to older patients, male patients, and those with B-cell malignancies treated with bendamustine, not only in preventing SARS-CoV-2 infection but also in ensuring prompt and active treatment when COVID-19 is contracted.

## Data Availability

The raw data supporting the conclusions of this article will be made available by the authors, without undue reservation.
