# Supplementary material for: Severe and/or prolonged COVID-19 in hematologic diseases: clinical implications before and during the omicron era
Source: Front Oncol. 2025 Nov 5;15:1687204. doi: 10.3389/fonc.2025.1687204 (PMC12626787; doi:10.3389/fonc.2025.1687204)
Supplement: Supplementary file 1 [file Table1.docx]

**Supplementary Table S1. Sequelae after COVID-19 treatment**

| **Post-COVID-19 sequelae** | **n=79 (8.5%)** |
| --- | --- |
| **Respiratory complication** | 57 |
| Intestinal pneumonia | 42 |
| Aspiration pneumonitis | 6 |
| Bacterial pneumonia | 5 |
| Pneumothorax | 3 |
| Pyothorax | 1 |
| **Non-respiratory infection** | 6 |
| Severe fatigue | 5 |
| Idiopathic thrombocytopenic purpura | 3 |
| Renal failure | 2 |
| Thrombotic disease | 2 |
| Gastrointestinal bleeding | 1 |
| Heart failure | 1 |
| Cacogeusia | 1 |
